# Pursuing the impossible: an interview with Tim Hunt

**DOI:** 10.1186/s12915-015-0164-y

**Published:** 2015-08-08

**Authors:** Tim Hunt

**Affiliations:** ᅟ, ᅟ

## Abstract

Tim Hunt took an undergraduate degree in Natural Sciences at Cambridge in 1964, and his PhD and subsequent work focussed on the control of protein synthesis until 1982, when his adventitious discovery of the central cell cycle regulator cyclin, while he was teaching at the Marine Biological Laboratory in Woods Hole, redirected him to the study of cell cycle regulation. From 1990 to his retirement Tim worked in the Clare Hall Laboratories of Cancer Research UK. He shared the Nobel Prize in Physiology and Medicine with Lee Hartwell and Paul Nurse in 2001, and talked to us about the series of coincidences that led him to the prizewinning discovery.

## How did you, as a biochemist, get interested in the cell cycle?

It was an accident basically. I suppose the key moment was a seminar in Woods Hole in 1979, when John Gerhart came one afternoon to tell us about his recent studies of MPF [maturation promoting factor], which I hadn’t heard of before. MPF was this magic factor — ‘factor’ because nobody really knew what it was — that catalysed oocyte maturation. Maturation is a complicated process, but at its heart is a cell cycle transition, the G2 to M transition as we’d now say. The factor, originally described a few years before in a beautiful paper by Yoshio Masui [1], was heat-labile and protease sensitive, so almost certainly a protein, and therefore probably an enzyme. The idea that there was an enzyme that could catalyse a cell cycle transition astounded me, because in my book enzymes would usually catalyse rather trivial and boring reactions and this was something quite spectacular.

**Fig. 1:**
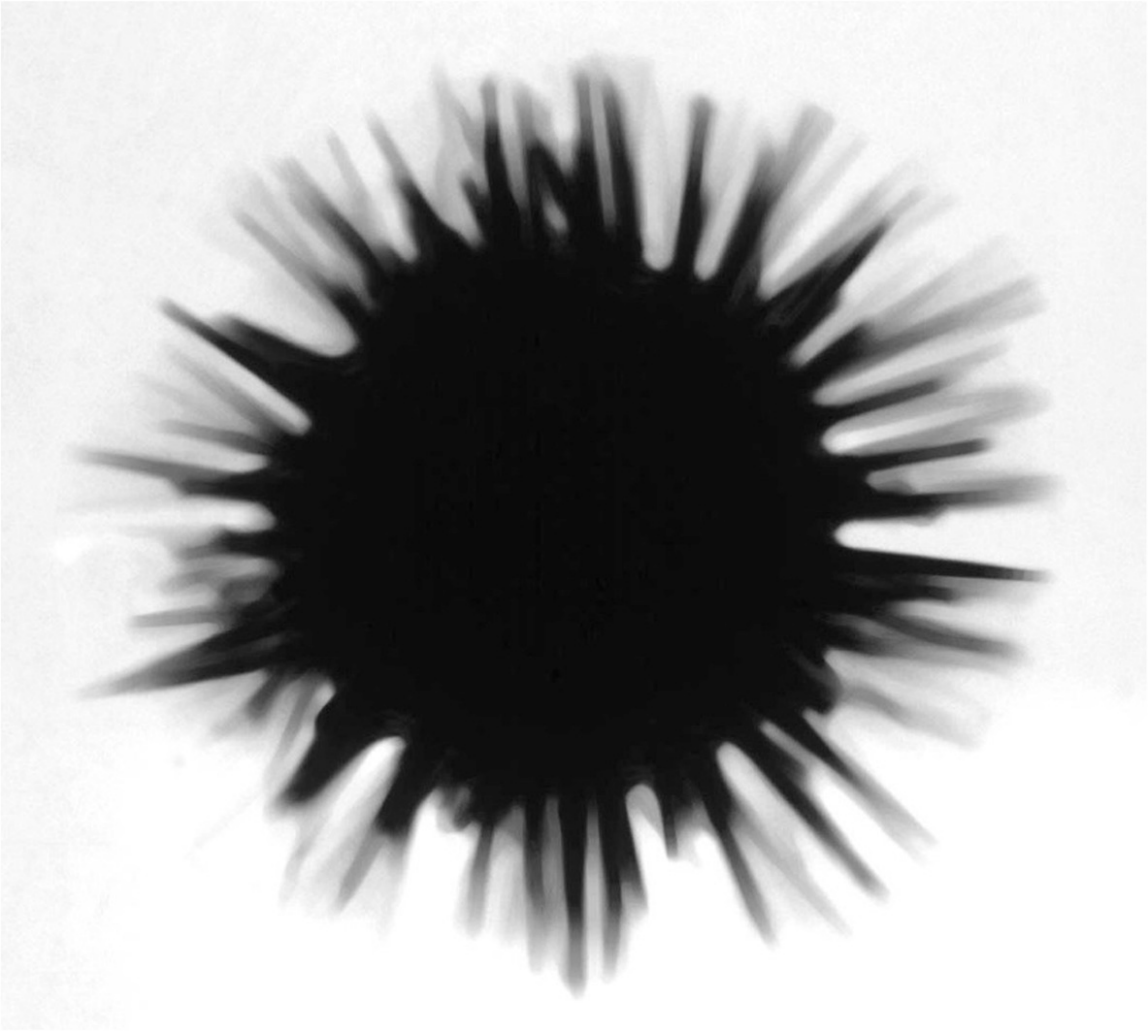
The sea urchin *Arbacia punctulata* in whose eggs cyclin was first discovered. This photograph was taken by Tim Hunt in his laboratory in the Marine Biological Laboratory in Woods Hole, Massachusetts

**Fig. 2:**
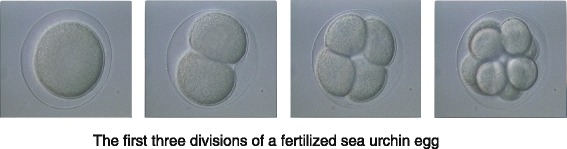
The first three divisions of a fertilized sea urchin egg

So I began to think about cell cycle control from that moment. I was working on the activation of protein synthesis in sea urchin (Fig. 1) and clam eggs at the time and we began to wonder why it was that — again something that had been known for a long time — fertilised sea urchin eggs needed new protein synthesis in order to divide: what were these proteins — we assumed there were several proteins — that they needed to divide? We knew they could synthesise DNA without new protein synthesis, but they couldn’t divide. Sometime around then a paper was published that showed that there was a critical period for each cycle where you had to make new proteins in order for the next division to take place. That didn’t strike anybody as unusual, because if you think about normal cells, they have to double in size, so of course they need to make new proteins. But sea urchin eggs don’t double in size, they actually halve in size at each division (Fig. 2) — and nor do clams’. So that’s really what triggered it.

I went to see a friend who was teaching himself how to microinject sea urchin eggs, to see whether sea urchin eggs contained MPF that you could assay either in sea urchins or frogs. We never got around to it, it was too difficult, and we weren’t really serious or asking the right questions. I’d gone to Woods Hole originally to study how protein synthesis was turned on at fertilization, but you couldn’t avoid noticing that after they had turned on protein synthesis, they divided and went on dividing. The idea that there might be an enzyme behind it was also fascinating. I thought, what a delicious problem, but I didn’t work on it because I had no entrée — I had my own fish to fry so to speak.

## So how did you come to work on cell cycle control?

**Fig. 3:**
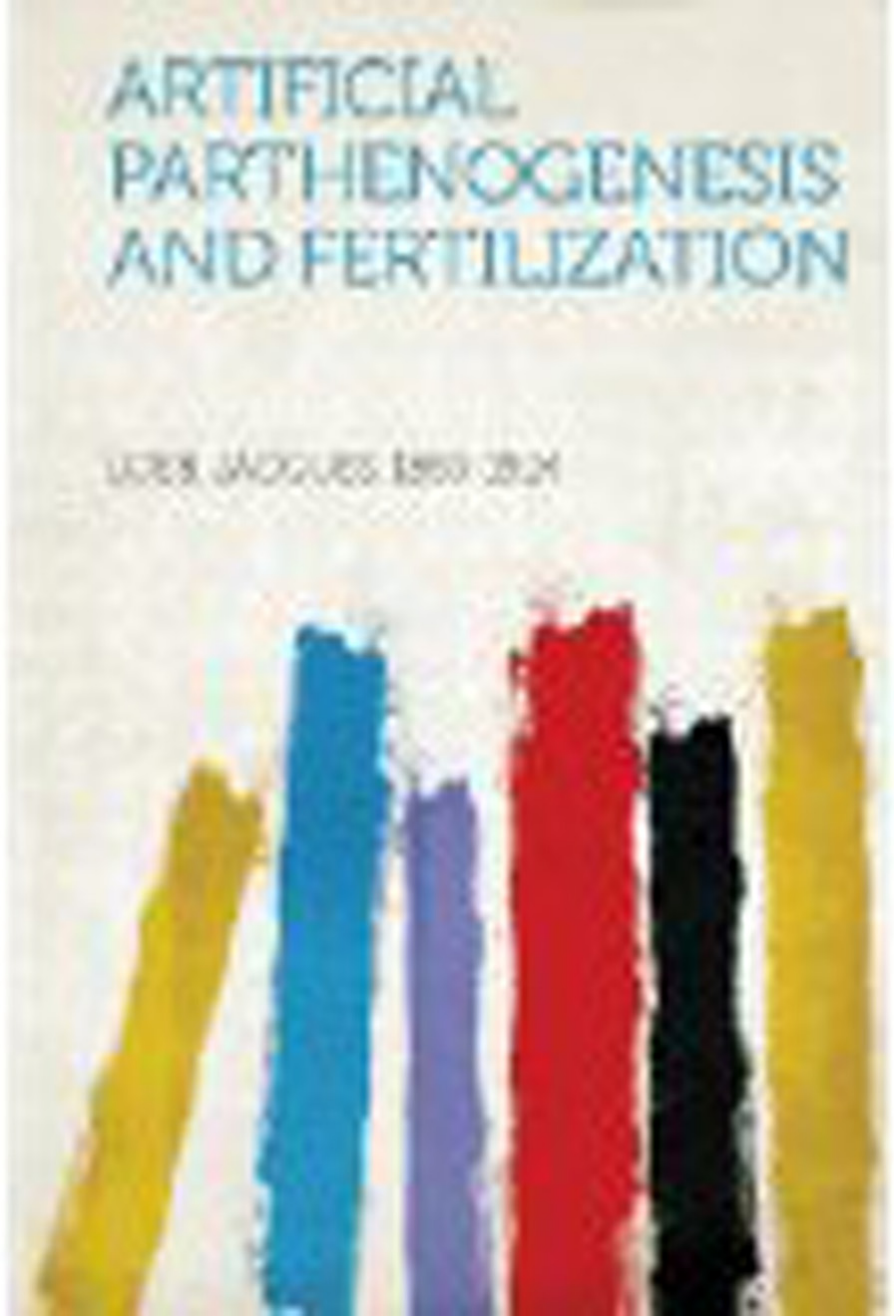
Jaques Loeb’s book *Artificial Parthenogenesis and Fertilization*

I think it’s quite funny really. For some reason, I’d come across the work of Jacque Loeb, who’d written a book called *Artificial Parthenogenesis and Fertilisation* [2] (Fig. 3). I think it was my religious upbringing that led me to enjoy doing experiments on the biochemistry of virgin birth. Loeb described experiments where things like dilute soap solutions or ammonia would cause the sea urchin eggs to activate and get going. At the time, in 1982, my main research problem was going very badly, so as a sort of afterthought — the formal part of teaching the course had stopped — I just idly wondered whether the patterns of protein synthesis in parthenogenetically activated sea urchin eggs was the same as or different from what happened when you fertilized them properly.

## So you had no deep intellectual reason for doing the experiment?

No. Not at all! There was some reason to think there might be a difference, because somebody had published a paper that said things went wrong in the parthenogenetically activated eggs. So I just did a simple experiment to compare normal patterns of protein synthesis with those in parthenogenetically activated eggs, and blow me down, when I developed the gel, which now hangs in the corridor of the lab where I used to work, one of the proteins — and it was one of the strongest proteins that was made early on — faded away at roughly the time the eggs divided. Then it came back again and got destroyed again when the eggs divided again. And I immediately realized that I had made a fantastic discovery. Proteins didn’t disappear in those days.

And this was reinforced by a second encounter with John Gerhart when I ran into him at the wine and cheese party that traditionally follows the Friday Evening Lecture at MBL [the Marine Biological Laboratory]. I asked him how things were going with MPF. He told me this electrifying result that he and Marc Kirschner had found. Although the first appearance of MPF did not require protein synthesis, the second appearance of MPF did. That exactly corresponded to what I’d seen — obviously if you’d got rid of a protein, to get it back you’ve actually got to synthesize it, and that very naturally explained why inhibiting protein synthesis in fertilized sea urchin eggs inhibited cell division. We’d seen this one protein go, but most of the proteins didn’t get destroyed when the sea urchin eggs divided — most of them just went on accumulating and accumulating. It was very easy and quick to show that it wasn’t a matter of rapid turnover and periodic synthesis — it was continual synthesis and occasional catastrophic proteolysis that was causing the sawtooth waves in cyclin levels, and that was basically it. I called the disappearing protein cyclin simply because of this behaviour, and wrote it up in a state of high euphoria, so much so that one of the reviewers said, ‘this is wild speculation based on faulty logic’. One of the other reviewers was Paul Nurse, who said ‘this is pretty interesting’, so I was lucky. I got a funny editor’s comment saying, ‘Dear Tim, the good news is that we will publish your paper, but the bad news is, in nothing like in its present form’. I had to rewrite it without having any new data whatsoever, because you couldn’t do any more experiments until the sea urchins became ripe again the following July. So it was a tricky situation. I somehow was able to satisfy the chap who thought it was illogical, but it’s not a paper I’m particularly proud of. You can see that my mind is actually somewhere miles and miles away from the main point [3].

## Do you think your paper would have survived the sort of criteria that journals are now increasingly espousing for reproducibility of research results?

No, I think not. If you tried to publish that kind of thing today, they’d want to know the precise molecular mechanisms of everything. When we went back the following summer, obviously the first thing that I cared about was repeating the experiment. I was worried by that time that I’d built such castles in the air that it might be mirage and wasn’t true, but fortunately it was. And then we could get stuck in and get going.

## What sort of castles had you built?

Well, I was pretty sure there was some sort of connection between MPF and cyclin. MPF was still just an activity at this stage. No one had any kind of molecular handle on it, and I didn’t dare even to hope there could be a direct connection. There were two problems with cyclin actually being MPF as I saw it at the time. One was that entry into mitosis is a rather abrupt, switch-like business, whereas accumulation of new protein is a rather linear business, so how do you turn a gradual increase in something into a sharp, switch-like response? The other thing that bothered me was the question of whether you could really make enough new enzyme in 15–20 minutes that it could catalyse a cell cycle transition? That seemed to me a bit unlikely. So we preferred the rather vague idea that cyclin might be a hypothetical anti-anti-MPF.

It took ages and ages to figure out what was really going on. Of course we now know that there is an elaborate regulatory machinery involving phosphorylation that helps to account for the switch-like response — but at that time we really had no information about what we were dealing with. We needed to clone and sequence the wretched thing. Luckily, there was absolutely no interest, and the cyclin paper was hardly cited at all for the next 5 years. Probably ‘wild speculation based on faulty logic’ was many people’s reaction. I was getting very sceptical looks from people and my pals in Cambridge: ‘Tim’s gone off his rocker’. Everybody says they now remember me showing them this gel and how excited I was, but obviously I wasn’t very good at explaining why. I wasn’t too good at articulating it, because really there wasn’t anything to articulate. I just knew it was right, but the details were totally unknown and unknowable actually at that stage.

**Fig. 4:**
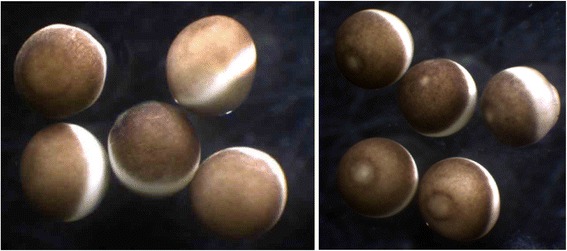
Frog oocytes before and after maturation. Oocytes (*left-hand side*) are surgically removed from the belly of a female frog and incubated in the presence of progesterone, which induces maturation. The white spot (*right-hand side*) that forms after a few hours marks the position of the second meiotic spindle, indicating completion of the cell-cycle transitions that occur at maturation

The next great advance came in the summer of 1986 at Berkeley, California. By that time, we knew there was more than one cyclin, though we didn’t know why, and Eric Rosenthal had cloned cyclin A from clams when he was a graduate student with my friend Joan Ruderman. Joan and her postdoc Katharine Swenson had done the brilliant experiment of injecting cyclin messenger RNA into frog eggs, which then matured [4]. That really put the cat among the pigeons. I wanted to repeat that for a start. We did it using RNA from *Urechis* eggs, a degenerate annelid worm from the mudflats of northern California that Eric was working on — and that worked, and then we wondered whether, if we made mRNA from mature frog eggs, whether that would work — and it did. John Gerhart’s technician Mike Wu had never ever seen an oocyte mature in response to injected messenger RNA, and he’d injected absolutely everything — he was the ace injector in the Bay Area — so we were tremendously excited. We went back to the lab at ten o’clock at night, and there the white spots were forming (Fig. 4)…it was such a simple experiment! So frog eggs contained mRNA that made frog eggs mature.

Then you really knew. Previously cyclin was a protein that came in weird organisms like sea urchins and clams, and nobody cares about sea urchins or clams, and then *Urechis*, and that’s even more obscure. But to have frog mRNA catalyse its own maturation. Wow! Tada! We were home and dry and knew we’d be able to find the cyclin mRNA in frogs. Jon Pines had been working with me on cloning cyclin from sea urchin eggs, and once he had a clone for sea urchin cyclin B, we screened frog cDNA libraries, and sure enough there were positives and we sequenced them and sure enough they all had the same or related sequences. We didn’t go after human cyclin because Jon graduated and went off to work with Tony Hunter with the idea of looking for the human cyclins. It was very quick and easy for him then to pick up that ball and run with it, which was very gratifying.

## At that point, you knew you were onto something that controlled the cell cycle, but you didn’t know how it did it?

We didn’t know how it worked, no, not at all. We were incredibly slow and stupid. I mean, there were some very clever people involved with this and I don’t think it ever crossed any of our minds that cyclins might be the activating subunit of a protein kinase, because the only precedent was the inhibitory subunit of protein kinase A. In retrospect, you can see that our thinking was incredibly constrained by existing paradigms.

## Did anyone know anything about regulation of the cell cycle?

Very little was known. The only people who knew a little bit were the yeast people, Lee Hartwell and Paul Nurse. But they were geneticists, very formal thinkers, not really interested in how things actually worked. So a very important moment came when Bob Booher sequenced *cdc13*. The Cdc genes encoded proteins that when mutated screwed up the cell cycle in yeast: the yeast cells just stopped dividing. Cdc13 was a perfectly kosher Cdc mutant in fission yeast, but with nothing to say that it was in any respect interesting or important. Bob famously presented his sequence at a seminar. In those days you put up the whole sequence with its translation, and Mark Solomon in the audience spotted that it was cyclin. He’d been looking for cyclin genes in yeast without much success, but he knew enough to recognise the now famous MRAIL sequence when he saw it, and then comparing clam cyclin A and sea urchin cyclin B sequences with Cdc13 clearly proclaimed it as a close relative of cyclin. That was it. When you mutated cdc13, the cell cycle stopped.

So that was an amazing and dramatic moment. Andrew Murray called me up from California and said ‘Cdc13 is cyclin’. I’d never heard of Cdc13 of course, being an ignorant biochemist, so it meant nothing to me, but I realised gradually. I was sworn to darkest secrecy. As ill luck would have it I had to go and visit Paul Nurse’s lab the very next week, and I couldn’t tell them about this important discovery. I kept asking, “Have you found anything that looks like cyclin?” “No, no, there’s nothing like cyclin”. Somebody was trying to sequence *cdc13* and it wasn’t going very well, so they hadn’t spotted it. By present day standards we were so amateurish and bumbling. I think Paul would say the same thing.

## Why hadn’t cyclins been discovered before?

That is kind of interesting I think, and just shows I was incredibly lucky, because in principle they could’ve been discovered at any time in the preceding 10 years. Brigid Hogan and Paul Gross studied patterns of protein synthesis in sea urchin eggs just before the one-dimensional SDS polyacrylamide gel had been invented, so they didn’t have the analytical tools. Unfortunately for the next person to look, Pat O’Farrell had just invented the two-dimensional gels, so anyone with any nous would run 2D gels. The problem with 2D gels is that it’s very difficult to run lots of them and get them reproducible, and anyway, cyclins don’t focus very well on 2D gels for some reason. I don’t really understand why not, but we tried for ages and could never see them. I was pretty good at running one-dimensional SDS gels, and it was only by doing this really dumb simple experiment that I saw it first.

So I was just the first lucky person who applied this simple technology to a simple problem, and happened to have had the right background in the form of tuition from John Gerhart to interpret what I saw more or less correctly, for the very first time. This was very unusual. Normally, discoveries are made by graduate students, but in this case it was me. One morning I didn’t know anything about the control of the cell cycle and the next morning I did. It was a good story, at least the way I reconstruct it in my mind. It really was a eureka moment, or eureka week maybe. I knew I’d discovered something really important because in those days, proteins did not just go away like that. They couldn’t just go away, it was theoretically impossible. I realise that really, if you want to win a Nobel prize, the thing to do is to do something which is theoretically impossible. Like, for example, sequence DNA, which I was reliably informed as a student was theoretically impossible, or solve the structure of the ribosome, which I was reliably informed as a student was theoretically impossible.
